# Prenatal Sex Hormone Exposure Is Associated with the Development of Autism Spectrum Disorder

**DOI:** 10.3390/ijms24032203

**Published:** 2023-01-22

**Authors:** Mengwei Li, Noriyoshi Usui, Shoichi Shimada

**Affiliations:** 1 Department of Neuroscience and Cell Biology, Graduate School of Medicine, Osaka University, Suita 565-0871, Japan; 2 United Graduate School of Child Development, Osaka University, Suita 565-0871, Japan; 3 Global Center for Medical Engineering and Informatics, Osaka University, Suita 565-0871, Japan; 4 Addiction Research Unit, Osaka Psychiatric Research Center, Osaka Psychiatric Medical Center, Osaka 541-8567, Japan

**Keywords:** sex bias, sex difference, sex hormone, testosterone, androgen, autism spectrum disorder, extreme male brain, female protective effect, prenatal environment, development

## Abstract

Sexual differentiation is a major developmental process. Sex differences resulting from sexual differentiation have attracted the attention of researchers. Unraveling what contributes to and underlies sex differences will provide valuable insights into the development of neurodevelopmental disorders that exhibit sex biases. Autism spectrum disorder (ASD) is a neurodevelopmental disorder that affects an individual’s social interaction and communication abilities, and its male preponderance has been consistently reported in clinical studies. The etiology of male preponderance remains unclear, but progress has been made in studying prenatal sex hormone exposure. The present review examined studies that focused on the association between prenatal testosterone exposure and ASD development, as well as sex-specific behaviors in individuals with ASD. This review also included studies on maternal immune activation-induced developmental abnormalities that also showed striking sex differences in offspring and discussed its possible interacting roles in ASD so as to present a potential approach for future studies on sex biases in ASD.

## 1. Introduction

Sexually dimorphic or sex-biased disorders are well known and have been attracting attention from researchers for decades. Males are more susceptible to neurodevelopmental disorders (NDDs) including autism spectrum disorder (ASD), attention-deficit hyperactivity disorder, and intellectual disability, but females are more prone to psychiatric disorders, such as anxiety disorders, depressive disorder, and post-traumatic stress disorder [1,2]. In fact, these disorders have shown sex differences in their prevalence and symptoms [1].

ASD is well known for its male predominance. ASD is a heterogeneous NDD characterized by deficits in social communication and restricted and repetitive behaviors and interests (RRBs) [3,4]. Although the male-to-female prevalence ratio of ASD has previously been reported as 4:1 [5], a meta-analysis conducted in 2017 showed that the true male-to-female ratio is likely closer to 3:1 owing to diagnostic gender bias [6]. Nevertheless, ASD is considered a typical male-biased disorder [7]. Therefore, identifying the underlying sex biases in ASD would be valuable for elucidating not only its etiology but also potential clinical interventions.

Unfortunately, owing to the complexity and heterogeneity of ASD, our understanding of sex biases in ASD remains limited, and many conflicting and elusive findings have been reported. In recent years, genetic studies have made great breakthroughs in identifying the causative and risk genes for ASD. As might be expected, studies have demonstrated that sex-associated genetic liability may contribute to sex biases in ASD [8,9]. Hypotheses, such as the female protective effect (FPE) theory, have attempted to explain sex biases in ASD from a genetic perspective.

To date, more than 1000 genes have been associated with ASD (https://gene.sfari.org (accessed on 13 December 2022)) [10,11,12]. ASD-associated genes are involved in various processes of fetal brain development, in particular neuronal differentiation, migration, and synaptogenesis [10,13]. For instance, one of the most frequently reported ASD genes, *SHANK3*, is a multidomain scaffold protein involved in the regulation of the structural organization of dendritic spines [14]. *FOXP1* is also frequently reported to be involved in social communication, cortical development, synaptic plasticity, and the regulation of ASD-associated gene expression [15,16].

However, a study estimated that genetic variants contribute to ASD in only 5–30% of patients [17]. Moreover, ASD is known to be caused by both genetic and environmental factors; therefore, genetic approaches would be insufficient, and studies that focus on environmental factors as well as the interaction between genes and the environment are also necessary.

As a result, an increasing body of evidence has shown that the environmental factors for ASD are also complicated and heterogeneous. For instance, maternal infection reportedly increases the risk of ASD in infants [18,19]. During pregnancy, bacterial, viral, or parasitic infections induce an immune response in mothers, i.e., maternal immune activation (MIA), which may subsequently lead to an increased risk of ASD in their offspring [19,20,21]. Maternal-derived proinflammatory cytokines produced by MIA cause impairments of the placenta itself and fetal brain development during pregnancy [19,21].

Studies on sex hormones have provided valuable insights into sex biases in ASD. Moreover, ASD may result from abnormal testosterone exposure and/or disrupted hormonal balance during prenatal development periods [22]. Hypotheses such as the extreme male brain (EMB) theory concerning prenatal testosterone exposure have been proposed to explain the sex biases in ASD. This review mainly focuses on the evidence of possible environmental factors for ASD and its male preponderance, i.e., the function of testosterone and other sex hormones during sex differentiation and the possible association between prenatal testosterone exposure and the development of ASD. In addition, we also discuss sex differences in MIA-induced abnormalities in offspring and their possible interrelation with ASD.

## 2. Body Development and Sexual Differentiation

**Figure 1:**
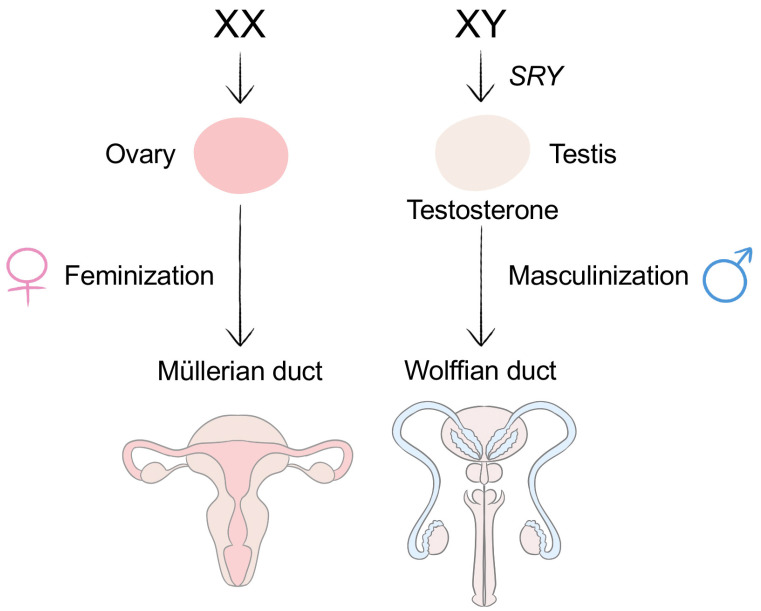
Sexual differentiation of the body. Sex is first determined by the pair of sex chromosomes a zygote contains: XX for females and XY for males. The absence or presence of the *SRY* gene on the Y chromosome determines whether the sexual differentiation process will be feminization or masculinization. For masculinization, the *SRY* gene directs the formation of the male-type gonad, testes. For feminization, since there is no Y chromosome and thus no *SRY* gene to activate the formation of the testis, an ovary is formed. The formed ovary, or the absence of the testis, promotes the Müllerian duct to further develop into female sex organs. For masculinization, the testes produce testosterone, and the development of the Wolffian duct is promoted to form male sex organs.

Sexual differentiation is an important process of human development. Both genetic and endocrine factors have a crucial impact on this process. Sex is determined the moment a female egg is fertilized by a male sperm and a zygote is formed. More specifically, the pair of sex chromosomes that a zygote contains, i.e., XX or XY chromosomes, determine an individual’s biological sex. This pair is contributed by the egg and sperm: the egg contributes one X chromosome and the sperm contributes either one X chromosome, resulting in female embryo development, or one Y chromosome, resulting in male embryo development. The pair of sex chromosomes in turn determines an individual’s gonad type, initiating a series of complex events of sexual differentiation in the body, including the formation of gonads and sex organs and the secretion of sex hormones, as well as sexual differentiation in the brain, which occurs later during fetal development (Figure 1).

Before sexual differentiation occurs, the gonads are bipotential, meaning that they can develop into either male-type or female-type sex organs. In the case of males, the initially undifferentiated gonads are activated by the *SRY* gene on the Y chromosome to form the male-type gonad, testes [23]. However, in females, the *SRY* gene is absent because the embryo has two X chromosomes. Consequently, the formation of the testes is not activated; instead, a series of genes on the autosomal and X chromosomes, including *WNT-4*, *DAX-1*, *FOXL2*, *COUP-TFII*, and *RSPO1*, activates the process for undifferentiated gonads to develop into ovaries [24]. Specifically, the absence of the testes promotes the development of the Müllerian duct, which is responsible for the development of female sex organs. Gonads that differentiate into ovaries do not secrete much sex hormone until puberty onset [25]. Once puberty begins, the ovaries start producing estrogen and progesterone, which further feminize the body by acting directly on the estrogen and progesterone receptors.

In males, the *SRY* gene on the Y chromosome encodes a protein called the testis-determining factor, which differentiates the bipotential gonads into the testes [26]. Once testes develop, androgens, such as testosterone and dihydrotestosterone, are secreted by the testes to promote and regulate the development of the male sex organs, brain, and the rest of the body. Testosterone promotes male differentiation by binding to androgen receptors to modulate gene expression related to male differentiation. In addition, the development of the Müllerian duct must be repressed, because it leads to the formation of female sex organs [27]. Instead, the Wolffian duct develops into male sex organs. The formation process of male sex organs is time-sensitive; it requires the surge in sex hormones to occur only within a specific time during development [28].

## 3. Brain Development and Sexual Differentiation

**Figure 2:**
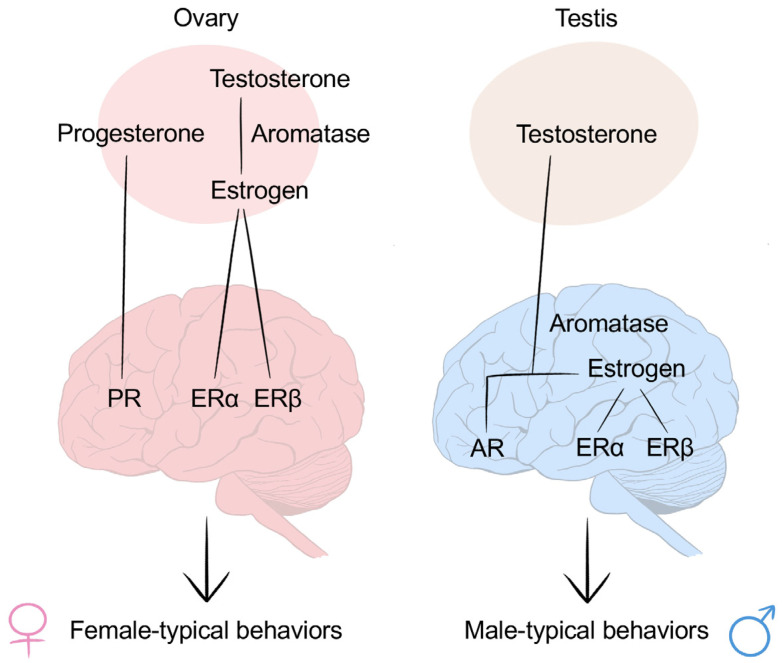
Sexual differentiation in the brain. The organizational–activational hypothesis is the classic review of sexual differentiation of the brain. The brain is organized by sex hormones secreted by the gonads. In females, the absence of the testes keeps the brain from being exposed to a high level of testosterone so that the brain will not be masculinized. Later, at the onset of puberty, the ovaries start to produce female sex hormones, including progesterone and estrogen, which bind to progesterone receptors and estrogen receptors (ERα and ERβ), respectively, and activate the brain to promote female-typical behaviors. In males, during the late gestation and early postnatal period, testosterone that is secreted by the testes binds not only directly to androgen receptors as testosterone, but also to ERα and ERβ after it is aromatized into estrogen, so as to masculinize and defeminize the brain. Once puberty begins, the masculinized brain is activated to promote male-typical behaviors.

Sexual differentiation occurs in both the body and brain. Similarly, the differentiation of the body and the differentiation of the brain results from the actions of activated genes and sex hormones, especially testosterone and its byproduct, estrogen. According to the organizational–activational hypothesis, the classical view of the effects of hormones on the brain, these sex hormones organize the brain into either the male- or female-type during the perinatal period. Subsequently, sex hormones continue to act on the brain to promote sex-specific reproductive behaviors [29] (Figure 2).

In females, without a high level of androgen exposure, the brain is feminized. Subsequently, during puberty, the sex hormones produced by the ovaries begin to activate female-specific reproductive behaviors.

In males, once the testes have formed, the initially bipotential brain is also exposed to androgens secreted by the testes. During the brain differentiation process, testosterone not only acts on androgen receptors but is also converted into estrogen to masculinize and defeminize the brain [30].

In humans, the third trimester of pregnancy is the time for fetal brain growth and development. During the last trimester, the brain rapidly grows. Brain size increases approximately five-fold [31]. Studies using structural and functional magnetic resonance imaging and postmortem examinations have revealed structural sex differences in the adult human brain. Human studies and their findings on sex differences are summarized in Table 1. On average, males have larger brain volumes and brain weights than females, but the proportional sizes of individual regions are similar [32].

Sex differences have also been reported in certain brain regions. Some areas of the brain, such as the straight gyrus [34], Wernicke’s area, Broca’s area [33], and hippocampus [35], are larger in the female brain than in the male brain. Furthermore, the splenium, the posterior region of the corpus callosum, reportedly has different shapes in males and females—females have a more bulbous shape and males have a more cylindrical shape [36]. In contrast, some other brain areas such as the amygdala [35], cerebellum [37], and preoptic area (POA) in the hypothalamus are larger in males than in females. The sexually dimorphic nucleus of the POA (SDN-POA), an area that is greatly involved in reproductive behaviors and regulation of sexual differentiation induced by sex hormones, shows significant differences between males and females [57].

Sex differences in the structural connectome have also been reported. Ingalhalikar et al. studied 949 youths using diffusion tensor imaging and discovered that the brains of females and males are optimized for different abilities [39]. They found that males have greater within-hemispheric connectivity, which facilitates the link between perception and coordinated action. In contrast, females have greater between-hemispheric connectivity, which integrates communication between analytical and intuitive processing modes. These results could provide some clues as to why males have better motor and spatial abilities and why females have better memory and social cognition skills.

## 4. Effects of Testosterone in Humans

Previous studies, by administering testosterone injections to ovariectomized but genetically female rodents immediately after birth and again at the onset of puberty or in adulthood, demonstrated that without the Y chromosome and male sex organs, testosterone alone is sufficient to masculinize the brain and behaviors of female rodents [58,59]. Testosterone has an influence not only on non-human animals but also on humans. Although manipulating sex hormone levels in humans is not possible for ethical reasons, researchers can use many other approaches to explore the impact of testosterone and its function in humans.

Similar to the testosterone surge that occurs in rodents, there is also an increase in the testosterone level in humans during prenatal development and early postnatal period [48], suggesting that testosterone may play a role in humans similar to that in rodents. A meta-analysis conducted in 2020 reported that baseline and changes in testosterone levels were positively associated with aggression in only men [60]. Other studies on testosterone have reported findings such as testosterone-related variants affecting facial features that are linked to sexual dimorphism in humans [49] and fetal testosterone levels influencing subsequent local gray matter volume of certain brain regions that are sexually dimorphic [50]. Furthermore, girls who are exposed to increased testosterone levels because of congenital adrenal hyperplasia are more likely to engage in male-typical play [27]. Taken together, although sexual differentiation and behaviors in humans involve several complicated factors in addition to testosterone, the sex hormone has critical functions in human development.

## 5. Sex-Biased ASD

Studies conducted on the effects of testosterone in humans have also elucidated sex-biased psychiatric and mood disorders. For instance, a meta-analysis conducted in 2021 found that prenatal testosterone exposure and serum testosterone concentrations were associated with ASD, attention-deficit hyperactivity disorder, addiction, and schizophrenia [61].

Sex biases have also been found in many cognitive and learning behaviors considered relevant to ASD, such as empathy tasks, social attention, and RRBs. On average, females score higher on empathy tasks than males, and individuals with ASD score the least, regardless of their sex [40,62]. A similar pattern was found in a social attention experiment, wherein females with ASD exhibited attention to social stimuli that was greater than that of males with ASD yet comparable to that of healthy males [41]. In contrast, RRBs were more increased in males with ASD than in females with ASD [42], which suggests that affected males demonstrate more severe ASD symptoms than affected females. Apart from the differences in behavior, differences in the brain and facial morphology have also been reported. By using three-dimensional photogrammetry, a study found that, compared with healthy controls, patients with ASD of both sexes displayed increased facial masculinity [63]. Another study investigated “brain maleness” in individuals with ASD symptoms and found that it is driven by brain size; individuals with ASD have larger brains than controls, which is congruent with the finding that males have larger brains than females [64].

Taken together, these sex-biased results demonstrate that the risk factors are more male-specific, if not androgen-induced. Moreover, because females with ASD also show male-specific characteristics, this further indicates that there may be some sex-related risk factors that are partly responsible for ASD. In fact, several previous studies have demonstrated that many ASD-like traits are related to testosterone and that, in general, the level of testosterone exposure is positively associated with attention-to-detail tasks [51] and RRB- [65] and ASD-like traits such as pretend play, joint attention, and social communication [52], among others. These findings provide crucial clues to the etiology of ASD as well as the causes and factors that might be responsible for the sex biases in ASD.

## 6. Testosterone Exposure and the EMB Theory

**Figure 3:**
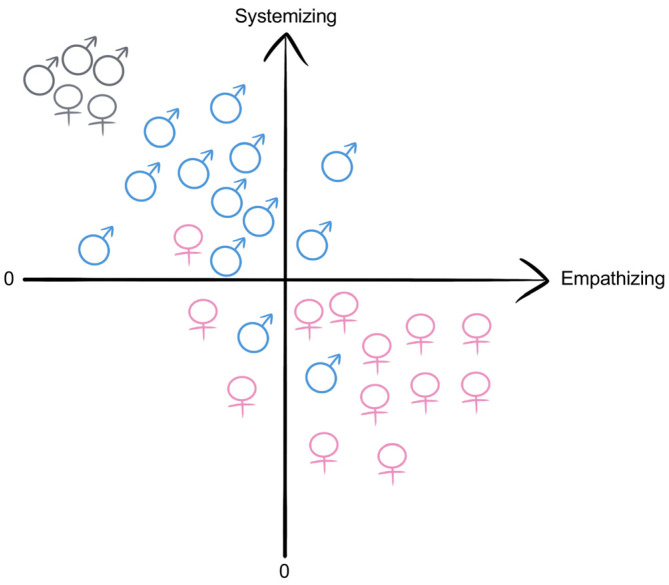
Empathizing and systemizing of EMB theory. According to EMB theory, a male-like brain results from elevated prenatal testosterone exposure. On average, males (blue-colored symbols) usually show a stronger interest in systems, whereas females (pink-colored symbols) tend to be more interested in empathy. The EMB theory states that, because individuals with ASD show extremely high interest in systems and low interest in empathy, they should fall into the top-left corner of the second quadrant (gray-colored symbols), i.e., the EMB.

To date, researchers have proposed many hypotheses to explain the sex biases in ASD and the effects of testosterone in individuals with ASD. One such hypothesis proposed by Baron-Cohen is the EMB theory. The EMB theory states that abnormally elevated levels of testosterone exposure during prenatal development may lead to a masculinized brain, which in turn may result in male-like cognition and behaviors, such as those mentioned above [53,66,67,68] (Figure 3). The EMB theory may account for the high male prevalence and male-specific characteristics in ASD, since prenatal development is an extremely crucial time period for the brain; thus, abnormal hormonal changes may have a great influence on the brain structure.

In humans, there are many occurrences that could lead to increased testosterone exposure during gestation. For instance, psychological stress correlates with testosterone concentrations in women [69], and maternal stress during pregnancy is reportedly associated with a higher ASD risk in offspring [70]. Furthermore, one of the most common androgen-related disorders, polycystic ovary syndrome (PCOS), can induce a hyperandrogenic environment in utero during pregnancy. 

PCOS is characterized by hyperandrogenism, ovulatory dysfunction, and polycystic ovarian morphology; according to an estimate, PCOS occurs in 5–20% of women of reproductive age [71]. Notably, increased testosterone levels have been reported in the majority of women with PCOS [72]. Furthermore, women with PCOS have higher testosterone levels during pregnancy [73], which could potentially constitute an environment of abnormally elevated testosterone exposure for the fetus. Owing to the nature of PCOS, it was thought that the children of women with PCOS might have a higher risk of ASD. Several studies have reported findings that support this hypothesis [74]. Furthermore, a study conducted by Palomba et al. provided evidence indicating that daughters born to mothers with PCOS might be affected more than sons; female (but not male) fetuses of women with PCOS were exposed to a higher level of testosterone in the amniotic fluid during the second trimester than those of healthy mothers [55]. The study also reported that the daughters of women with PCOS showed significantly increased ASD traits later in life. However, a more recent study did not find an increased risk of ASD in female children of women with PCOS; instead, the study found that the first-born children of these women had 35% increased odds of developing ASD [56]. These findings from clinical reports are valuable for studying the relationship between prenatal testosterone exposure and sex biases in ASD, if not in other male-biased disorders (Table 1).

Most of the findings on testosterone and ASD have been provided by studies that mainly investigated the association between elevated testosterone concentration and ASD-like behaviors or alterations in the brain. The role of testosterone in susceptibility to ASD is still poorly understood. A recent study conducted by Yagishita-Kyo et al. using *Octodon degus* investigated neurexin and neuroligin [75], which are proteins that play crucial roles in regulating social behaviors [76]. Previous studies have identified a variety of mutations in *NLGN* and *NRXN* in individuals with ASD [77,78]. In their study, Yagishita-Kyo et al. found that testosterone interrupts intercellular neurexin–neuroligin binding by directly binding to neurexin. Since neurexin and neuroligin are single transmembrane proteins that are localized at pre- and post-synapses, respectively, the interruption caused by testosterone may result in abnormal synaptic formation, which leads to ASD development.

Studies have not only focused on the direct effects of testosterone but also on its metabolite, estradiol, and the estrogen receptor (ER). One study determined that the expression of aromatase and *ESR2* mRNA and its protein are significantly decreased in the brains of individuals with ASD [45]. ERβ plays an important role in neurodevelopment and in mediating behaviors such as anxiety, fear response, and learning [79]. Another study by Sarachana et al. demonstrated that the ASD candidate gene *RORA* that encodes retinoic acid-related orphan receptor alpha is differentially regulated by male and female sex hormones through their respective receptors [46]. RORA is a hormone-dependent transcription factor, and one of its transcriptional targets, aromatase, is responsible for converting testosterone to estrogen. In their study, they showed that RORA and aromatase were reduced in the frontal cortex of individuals with ASD, indicating that testosterone might not have been properly converted into estrogen, which may eventually lead to elevated levels of testosterone in individuals with ASD. In other words, it is speculated that the association of elevated testosterone levels with a higher risk of ASD may result from a deficiency of RORA and aromatase. In addition, since testosterone levels are much higher in males and estrogen levels are higher in females, the reduction in aromatase may partially explain the sex biases in ASD.

## 7. Animal Models of Elevated Prenatal Testosterone Exposure

Despite the findings on the relationship between prenatal testosterone exposure and ASD risk, there are still many limitations to clinical studies owing to ethical considerations. For instance, it would be impossible to identify areas that have been altered in the brain or body by elevated prenatal testosterone exposure.

The effects of prenatal testosterone exposure on the brain have been investigated by manipulating prenatal hormone levels in rodents. Studies conducted using animal models are summarized in Table 2. Previous studies have reported that prenatal exposure to testosterone results in masculinized morphology and behaviors, such as increased anogenital distance [80], altered aggressive behaviors [81], and male-like mating behaviors [59] in female mice and larger SDN-POA in female rat brains [38]. Moreover, a study also found that prenatal testosterone exposure induced increased density and abnormal morphology of dendritic spines in mice [82], which is consistent with the findings of increased spine densities in individuals with ASD [47], indicating that elevated prenatal testosterone exposure may cause deficits in synaptic development and hence a higher risk of ASD.

However, treating adult female mice with testosterone had only slight and transient effects [83], suggesting that exposure to testosterone must occur as early as before the end of puberty to have permanent changes in the brain structure; for testosterone to induce ASD-like behaviors, prenatal testosterone exposure may have more impact than postnatal exposure.

## 8. Sex Differences in MIA

Besides treating rodents prenatally, animal models of early-life immune activation have also provided clues for the underlying etiology of ASD from an immune-based perspective. MIA can alter fetal brain development and lead to ASD-like behaviors in offspring [84,85] (Table 1 and Table 2).

Moreover, a large body of evidence has shown that male offspring are more affected by MIA than female offspring. For instance, by exposing mice to polyinosinic-polycytidylic acid prenatally, Haida et al. reported in their study that although both male and female pups showed delayed development, reduced social behavior, increased immobility in novel environments, and decreased motor/exploratory activity, motor coordination deficits were only observed in male pups [20]. In the same study, they found reduced cell numbers in the lateral parts of the cerebellum, its vermis, and in the motor cortex of male offspring, whereas in females, cell reduction was observed only in the paramedian lobule of the cerebellum.

In summary, MIA may have abnormal effects on fetal development and induce a higher risk of ASD in the offspring; the abnormal effects appear to be influenced by the biological sex of the fetus through an unknown underlying mechanism. This pattern, as mentioned previously, has also been observed in individuals with ASD, i.e., men are more affected than females in terms of risk of ASD and severe symptoms.

Many theories have been proposed to explain these sex biases in neurodevelopmental alterations, ASD, and other NDDs. For instance, differing thresholds for ASD under a liability threshold model, which is the predominant model of the FPE theory, states that females with ASD carry a higher number of copy number variants and single nucleotide variants than males with ASD [88].

In addition, there are also studies reporting that the risk of being diagnosed with ASD is greater for relatives of females with ASD than relatives of males with ASD [43]. These findings suggest that females seem to require a higher mutational burden to meet the diagnostic threshold for ASD than men, explaining “protective” in the FPE theory. However, although males and females exhibit different levels of severity in behaviors and/or symptoms, it may be incorrect to assume that females are attenuated versions of males.

Using bacterial lipopolysaccharides, Braun et al. examined MIA-induced alterations in fetal brain development and the related effects in female and male mice [86]. They found that, while various abnormalities were observed in both sexes, the abnormalities were distinct, if not opposite in some cases, between male and female mice. These findings indicate that the mechanism of MIA may be completely different in female offspring than in male offspring. Provided that this is the case, the assumption that being female is “protective” in the FPE theory may need to be reconsidered. Studies on females with ASD and ASD-like behaviors and alterations in the female brain using animal models should not regard females as attenuated versions of males. Instead, they should focus on female-specific abnormalities that may have been neglected because they were not observed or were different in males.

## 9. Discussion

Owing to the complex factors involved, the exact causes and underlying mechanisms of ASD remain unelucidated. Nonetheless, results from studies on the sexual differentiation process and the function of sex hormones demonstrated that sex hormones, especially prenatal testosterone exposure, play a significant role in the development of ASD. Moreover, after decades of efforts to study the effects of testosterone, in recent years, an increasing number of studies have started to investigate not only testosterone but also its metabolites and receptors, as well as the genes that regulate their expression. 

For instance, there have been studies suggesting that testosterone per se does not affect the body and brain development and behavioral alterations; instead, androgen or estrogen receptors to which testosterone binds are responsible [89]. Hodosy et al. demonstrated that the effects of endogenous testosterone are blocked when rats are treated with a competitive inhibitor of the androgen receptor, supporting the hypothesis that the effects are not directly caused by testosterone. In fact, there is an increasing number of studies focusing on the function of androgen receptors and the metabolites in ASD [54,87,90]. Fetal development, sexual differentiation, and ASD development involve numerous factors rather than testosterone alone. It would be inappropriate and misleading to focus only on understanding the function of one sex hormone.

Finally, future studies may consider ASD or ASD-like behaviors in females and males independently, rather than comparing female and male ASD phenotypes. As mentioned previously, it is highly possible that women with ASD have a completely distinct set of alterations and abnormalities from males with ASD. An increasing number of studies have shown that females with ASD exhibit different traits than males with ASD [44,91]. Furthermore, clinical studies have also reported that, owing to different ASD traits and camouflaging, females with ASD tend to be diagnosed much later, if ever, than males with ASD [92,93]. For future research on sex differences and biases in ASD, ASD-like behaviors, abnormalities, and alterations occurring in females should be treated independently from those in males.

## Figures and Tables

**Table 1:** Summary of human studies on sex differences and ASD.

Focus		Subjects	Phenotype	Ref
Sex differences in brain morphology	Sex differences in the absolute and proportional volumes of the main language-associated regions of the cerebral cortex	21 postmortem brains (10 males, 49–86 y; 11 females, 46–92 y)	Larger volume of the superior temporal cortex and the cortical volume fraction of the Broca’s area in females	[33]
	The cortical sulci formation in the preterm newborn brain	35 preterm newborns (16 females, 19 males; 25.6–35.6 w)	Lower cortical surface, smaller cortex, and white matter volumes were observed in females	[31]
	The relationship between the structure of the ventral frontal cortex and social cognition in female and male	60 healthy, right-handed Caucasian participants (30 males, 18–50 y; 30 females, 18–50 y)	Larger SG in females, femininity correlated with greater SG gray matter volume, and surface area and better performance on the IPT	[34]
	Normal sexual dimorphisms of cortical and subcortical brain regions	48 participants from Boston, the United States (27 males, 39.1 ± 12 y; 21 females, 36.3 ± 10.5 y)	Larger volumes of frontal and medial paralimbic cortices in females, larger volumes of frontomedial cortex, the amygdala, and hypothalamus in males	[35]
	Sex differences in the corpus callosum	246 midsagittal images were selected out of approximately 1000 sets of MRIs scanned at four southern California MRI centers	Splenium was bulbous-shaped in females, tubular-shaped in males	[36]
	Age and sex differences in the cerebellum and the ventral pons	190 participants (77 males, 45.74 ± 16.90 y; 113 females, 47.09 ± 16.17 y)	Age-related reduction in the cerebellar hemispheres and the cerebellar vermis; the volume of the cerebellar hemispheres, the vermis, and the ventral pons were larger in males	[37]
	Sex differences in SDN-POA	30 postmortem human brains (13 males and 17 females, 10–93 y)	The volume of the SDN-POA was 2.2 times as large in males than in females and contained 2.1 times as many cells	[38]
	Sex differences in the structural connectome	949 participants (428 males, 521 females)	Greater within-hemispheric connectivity in males; between-hemispheric connectivity in females	[39]
ASD development and sex differences in ASD and ASD-like behaviors	The prevalence and characteristics of ASD	Children aged 8 years in 2018 whose parents or guardians lived in 11 ADDM Network sites in the United States	The overall ASD prevalence estimate was one in 44 children aged 8 years in 2018	[7]
	The association of genetic liability and sex biases in ASD	970 Caucasian families (2028 individuals) with at least two related ASD individuals from the ten AGP sites in North America and Europe	Lower repetitive behavior scores in females; higher scores in males from female containing families that had higher repetitive behavior scores	[8]
	ASD individuals and sex differences in the Empathy Quotient score	90 adults with AS/HFA (65 males, 25 females, 15.4–59.9 y); 71 males (17.4–69.6 y); 126 females (17.7–73.0 y)	Adults with AS or HFA scored significantly lower on the EQ; in a general population, females scored higher than males.	[40]
	Sex differences in attention to social stimuli	74 school-aged males and females with ASD (23 males, 19 females) and without ASD (16 males, 16 females)	ASD females attended more to faces in the socially lean condition; ASD males attended less to faces regardless of social context	[41]
	Sex differences in restricted and repetitive behaviors and interests in ASD youths	615 youth with ASD (507 boys, 108 girls, 3–18 y)	Increased self-injurious, compulsive, restricted, and insistence on sameness behaviors in females; stereotyped and restricted behaviors in males	[42]
	To determine if the risk in these families follows a female protective model by assessing recurrence patterns in multiplex families	12,260 individuals from 2278 families acquired from Autism Genetics Resource Exchange	Higher recurrence in males; interbirth interval negatively correlated with ASD recurrence that is driven by children in male-only families	[43]
	To examine whether females with ASD would display better social skills than males with ASD on a test of friendship and social function	101 participants in total; 50 ASD adolescents (25 males, 25 females), 51 typically developing participants (26 males, 25 females)	Females demonstrated higher scores on the friendship questionnaire than males	[44]
	Sex differences in the expression of RORA	Human neuroblastoma cells SH-SY5Y	Sex differences were observed in the expression of Rora/RORA and its transcriptional targets in the human brain	[9]
	Dysregulation of ERβ, aromatase, and ER co-activators in the middle frontal gyrus of ASD individuals	13 postmortem middle frontal gyrus tissues of ASD and 13 control subjects	Decrease in ERβ mRNA expression in the middle frontal gyrus of ASD subjects; a reduction in CYP19A1 mRNA expression was observed in ASD subjects	[45]
	Differences in male and female hormones in the regulation of RORA, a novel candidate ASD gene	The human neuroblastoma cells SH-SY5Y	Male and female hormones differentially regulate RORA	[46]
	Dendritic spines on Golgi-impregnated cortical pyramidal cells in the cortex of ASD subjects and age-matched control cases.	Cortical tissue samples were acquired from 10 postmortem ASD males and 15 male control subjects	Greater spine densities were observed in ASD subjects	[47]
Effects of testosterone or other sex hormones	The differences in reproductive hormone levels and their biological effects between full-term and preterm infant boys	25 full-term and 25 preterm boys (gestational age 24.7–36.6 w)	Postnatal HPG axis activation in infancy is increased in preterm boys and associated with faster testicular and penile growth	[48]
	The effects of testosterone-related genetic variants on facial morphology	7418 individuals belonging to three separate cohorts: 2297 participants (3–40 y, Western-European descent); 1555 participants (18–83 y; European descent); 3566 participants (14–17 y, Western–European descent)	Two intronic variants of SHBG, rs12150660 and rs1799941, showed an effect on mandible shape; Rs8023580 showed an association with the total and upper facial width-to-height ratios	[49]
	The effects of fetal testosterone on organizing the human brain for expression of sexual dimorphism later in life	28 boys (8 –11 y); 101 males (8–11 y), and 116 females (8–11 years) from the NIH Pediatric MRI Repository	Gray matter volume in RTPJ/pSTS was greater in males, positively predicted by fetal testosterone; gray matter volume in PT/PO and plOFC was greater in females, negatively predicted by fetal testosterone	[50]
	Effects of fetal testosterone on visuospatial ability in children	64 children (35 boys, 29 girls, 7–10 y)	Fetal testosterone was a significant predictor for Embedded Figures Test scores in both boys and girls	[51]
	The association of fetal testosterone with ASD traits in children	129 participants (66 boys, 63 girls)	Boys scoring significantly higher in the Q-CHAT	[52]
	The association of fetal testosterone and ASD traits	235 (118 boys, 117 girls)	Fetal testosterone levels were positively associated with higher scores on the CAST and the AQ-Child	[53]
	The relationship between parameters of androgenicity (plasmatic testosterone levels and androgen receptor sensitivity) and hyperactivity in boys	60 ASD boys (3–15 y)	No association between actual plasmatic testosterone levels and hyperactivity symptoms; the number of CAG triplets was negatively correlated with hyperactivity symptoms	[54]
The effects of MIA or PCOS on offspring	The association of MIA and ASD development in the offspring	114,500 children born in 1999–2009 from The Norwegian Mother and Child Cohort Study	Prenatal fever was associated with increased ASD risk	[18]
	The risk of developing PDDs in the offspring of women with PCOS	30 pregnant PCOS patients with hyperandrogenemia and 45 other pregnant healthy women	Children of PCOS patients scored higher on the AQC; daughters of PCOS patients scored higher on communication and attention switching	[55]
	The risk of developing ASD in the offspring of women with PCOS	971 women with autism were included in Study 1; 26,263 women with PCOS phenotype were included in Study 2; 8588 children of women with the PCOS phenotype in Study 2 who had first-born children linked in the database	Increased prevalence of PCOS in women with ASD and elevated rates of ASD in women with PCOS	[56]

ADDM: the autism and developmental disabilities monitoring, ASD: autism spectrum disorder, AGP: autism genome project, MIA: maternal immune activation, SG: straight gyrus, MRI: magnetic resonance imaging, SDN-POA: the sexually dimorphic nucleus in the preoptic area of the hypothalamus, HGP: hypothalamic-pituitary-gonadal, RTPJ/pSTS: right temporoparietal junction/posterior superior temporal sulcus, PT/PO: planum temporale/parietal operculum, plOFC: posterior lateral orbitofrontal cortex, AS: Asperger’s syndrome, HFA: high-functioning autism, Q-CHAT: the quantitative checklist for autism in toddlers, CAST: childhood autism spectrum test, AQ-Child: child autism spectrum quotient; PCOS: polycystic ovary syndrome, AQ-C: the autism-spectrum quotient child, ER: estrogen receptor, RORA: retinoic acid-related orphan receptor-alpha, IPT: interpersonal perception task, EQ: the empathy quotient.

**Table 2:** Summary of animal studies on sex differences and ASD-like phenotypes.

Focus		Species and Strain	Method	Phenotype	Ref
Effects of T or other sex hormones	Effects of neonatal androgen exposure on ovulation, sexual receptivity, and aggressive behaviors in female mice	Swiss Webster mice	Either 100μg TP, 100μg T, or 100μg AD on PN0–3	Females given either TP, T, or AD neonatally were anovulatory; more aggression in females given either TP or T neonatally	[58]
	Effects of prenatal T exposure on morphology and later behavior	Rockland Swiss albino mice	Either 1 mg T, 1 mg DHE, 1 mg PRO, or 1 mg PREG *s.c.* on E11–17 (1 mg/0.1 m)	Prenatal T, DHE, or PRO exposure increased anogenital distance of female mice	[80]
	Effects of prenatal T exposure on morphology and aggressive behavior	Female Rockland Swiss mice	1.5 mg TP *s.c.* on E12–16	Prenatal T exposure increased aggression in female offspring	[81]
	Effects of pre- and post-natal T exposure on differentiation of SDN-POA	Sprague Dawley rats	2 mg TP or DES *s.c.* from E16 until delivery	Larger SDN-POA in female rats treated with T propionate or diethylstilbestrol	[38]
	Effects of prenatal T exposure on dendritic spines	Mice expressing YFP in layer 5 pyramidal neurons (C57BL/6J background)	0.1 mg TP *s.c.* on E16–18	Instability and excess density of dendritic spines were observed in prenatally T-exposed mice	[82]
	Effects of T on the persistence of food searching	64 mice (48 males, 16 females)Half of the mice were BALB/c strain, and the other half were ‘porton’ mice	7.5 mg T enanthate	T produced increased persistence in castrated mice	[83]
	To establish degus as a highly socialized animal model and the effects of T on sociality-related genes	Octodon degus	-	Neurexin and neuroligin in degus brain are highly conserved to that of human sequences; T directly binds to degus neurexin and intercepts intercellular neurexin–neuroligin binding	[75]
Effects of MIA induced by poly(I:C) or LPS	Sex differences in behavioral deficits and neuropathology of ASD	C57BL/6J mice	poly(I:C) (20 mg/kg) *i.p.* on E12.5	Reduced social interactions and motor development and coordination deficits; reduced numbers of Purkinje cells in the cerebellum were more widespread in males	[20]
	Effects of prenatal allergen exposure on sexual differentiation, social, and sexual behaviors	Sprague Dawley rats	LPS (200 μ/kg) *i.p.* on PN0; Prior to pregnancy: 1 mg valbumin (4 mg/mL) subcutaneous injection; after 2 weeks: 1 mg ovalbumin-alum adjuvant injection	Increased mast cell and microglia activation in the neonatal brain; an increase in male-typical mounting behavior in adult females	[30]
	The mechanism by which MIA causes long-term behavioral deficits in the offspring	Female C57BL/6J mice; IL-6 KO mice (C57BL/6J background)	Cytokine injection: Either 5 μg carrierprotein-free recombinant mouse IL-6 or carrier-free recombinant mouse IFNγ *i.p.* Cytokine blocking: 20 mg/kg poly(I:C) *i.p.*	IL-6 caused prepulse inhibition and latent inhibition deficits in the adult offspring; MIA in *IL-6* KO mice does not result in several behavioral changes	[84]
	Effects of MIA and EIA on ASD-like behaviors and neuroimmune function	C57BL/6J mice	20 mg/kg poly(I:C) *i.p.* on E12.510 mg/kg LPS on PN9	EIA-produced sex-specific behavioral effects and immune responses in the brain	[85]
	Sex differences in immune-driven alterations in fetal brain development and related outcomes in female and male mice	C57BL/6J mice	30 or 60 µg/kg LPS *i.p.* on E12.5	A unique set of vulnerabilities and developmental consequences only in females	[86]
Studies using mutant mice and others	Effects of *Mdga2* haploinsufficiency on behavioral phenotypes related to ASD	*Mdga2* null mice	-	Increased excitatory synapses and function, altered LTP, and ASD-like phenotype	[76]
	Effects of increased anxiety on synaptic plasticity in ERβ-deficient mice	*ERα* and *ERβ* mutant mice	-	Increased anxiety, reduced threshold for the induction of synaptic plasticity in the amygdala in *ERβ* mutant females	[79]
	Effects of prenatal VPA exposure on AR	Male and female Wistar rats	600 mg.kg VPA *i.p.* on E12	VPA exposure alters AR expression in the postnatal developing cerebellum in both male and female rats	[87]

PN: postnatal, TP: testosterone propionate, T: testosterone, AD: androstenedione, LTP: long-term potentiation, DHE: dehydroepiandrosterone, PRO: progesterone, PREG: pregnenolone, DES: diethylstilbestrol, SDN-POA: the sexually dimorphic nucleus in the preoptic area of the hypothalamus, YFP: yellow fluorescent protein, poly(I:C): polyinosinic-polycytidylic acid, LPS: lipopolysaccharide, MIA: maternal immune activation, EIA: early life immune system activation, KO: knockout, IL-6: interleukin-6, IFNγ: interferon γ, VPA: valproic acid, AR: androgen receptor.

## Data Availability

Not applicable.
